# Silicone Fiducial Markers Improve Precision in Uveal Melanoma Radiation Therapy

**DOI:** 10.3390/cancers17020189

**Published:** 2025-01-08

**Authors:** Svenja Rebecca Sonntag, Olaf Wittenstein, Oliver Blanck, Jürgen Dunst, Stefan Huttenlocher, Melanie Grehn, Maximilian Busch, Dirk Rades, Ayseguel Tura, Salvatore Grisanti

**Affiliations:** 1 Department of Ophthalmology, University of Lübeck, University Medical Center Schleswig-Holstein, Campus Lübeck, 23562 Lübeck, Germanyayseguel.tura@uni-luebeck.de (A.T.);; 2 Department of Radiation Oncology, Christian-Albrechts University of Kiel, University Medical Center Schleswig-Holstein, Campus Kiel, 24105 Kiel, Germany; 3 Saphir Radiochirurgie Zentrum Norddeutschland, 24105 Kiel, Germany; 4 Department of Radiation Oncology, University of Lübeck, University Medical Center Schleswig-Holstein, Campus Lübeck, 23562 Lübeck, Germany; dirk.rades@uksh.de

**Keywords:** fiducial markers, uveal melanoma, radiotherapy, imaging, tumor delineation

## Abstract

**Objectives**: Accurate target definition, treatment planning and delivery increases local tumor control for radiotherapy by minimizing collateral damage. To achieve this goal for uveal melanoma (UM), tantalum fiducial markers (TFMs) were previously introduced in proton and photon beam radiotherapy. However, TFMs cause pronounced scattering effects in imaging that make the delineation of small tumors difficult. The aim of this study was to evaluate silicone fiducial markers (SFMs) for the guiding of stereotactic radiosurgery (SRS) for UM. **Methods**: In this retrospective interventional pilot case series, three patients with small UMs 3 mm or less in tumor thickness and ≤10 mm in largest basal diameter received silicone fiducial markers. The fiducial markers were punched out (3 mm) from conventional silicone encircling bands for buckle surgery. The markers were sutured onto the sclera at the tumor margins according to the use of TFMs. MRI and CT images were used for the localization of the tumor and the markers before robotic-guided SRS. **Results**: The silicone fiducial markers were punched out easily from the original band, better to handle than TFMs and easy to suture onto the sclera. They could be visualized in both MRI and CT, but were more visible in CT. In the absence of scattering effects, both the markers and thus the tumor boundaries could be clearly delineated. **Conclusions**: This is the first report that introduces fiducial markers intraoperatively shaped from conventional silicone encircling bands usually used for retinal detachment surgery. The SFMs allow more accurate tumor delineation, resulting in the more precise planning and administration of SRS when compared to TFMs. This simple modification has a major impact on a well-known treatment approach.

## Simple Summary

Radiotherapy with protons or photons is an indispensable treatment option for large and/or central tumors. However, radiotherapy can lead to varying degrees of radiation damage. Therefore, it is necessary to optimize treatment planning. Tantalum fiducial markers have been used for decades, but they lead to scattering artifacts and dose perturbations. This study evaluated the advantages and feasibility of silicone fiducial markers in radiosurgery treatment planning for uveal melanoma. Our results show that the absence of scattering and dose perturbation makes them an attractive alternative to tantalum fiducial markers.

## 1. Introduction

Radiotherapy for uveal melanoma (UM) with protons or photons is the treatment of choice for central tumors that are difficult to access for radioactive plaques, for tumors that are too large for brachytherapy and, depending on the surgeon, also for neoadjuvant radiation prior to resection [1,2,3,4,5,6,7,8,9,10,11,12,13,14].

However, radiation therapy is a double-edged sword that aims to destroy the malignancy but may cause substantial damage to surrounding healthy tissues. Precise target definition, treatment planning and administration of radiotherapy are therefore important to achieve an efficient tumoricidal effect with low radiation-induced toxicity.

In order to achieve this goal, tantalum fiducial markers (TFMs) were introduced to highlight the margins of the tumor during MRI (magnetic resonance imaging) and CT (computer tomography) and for radiation beam guidance during treatment delivery. TFMs were introduced in 1977 by Gragoudas et al., who realized the need for higher-precision techniques, especially for small UM [15]. Since then, several studies demonstrated the successful use of TFMs during proton beam irradiation. Up to five TFMs are fixed on the scleral surface at the tumor borders previously delineated by transillumination or fundoscopy. The delineation of the tumor with the marker positions thereafter helped in the planning of the target volume for irradiation.

**Figure 1:**
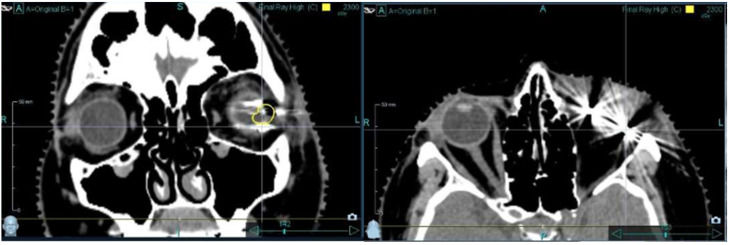
CT images of tantalum fiducial markers. Tantalum fiducial markers show huge scattering artifacts, which make the bulbus, and therefore, also the tumor, barely visible in some scans.

Nevertheless, the metallic TFMs have some disadvantages. These induce scattering artifacts in CT scans and X-rays and hence increase the uncertainty regarding the dose distribution of the radiation treatment. The artifacts also reduce the imaging quality and hinder the precise delineation and target localization of the tumor during treatment (Figure 1). In order to reduce these effects, others have already looked for alternative materials, but none of them have been successfully introduced into routine use until today [16,17,18,19].

Searching for an alternative material other than tantalum, we noted that silicone encircling bands, already in use for decades in retinal detachment surgery, are visible in CT scans.

The aim of this feasibility study was therefore to test silicone clips prepared from conventional encircling bands as fiducial markers for radiation therapy in patients with uveal melanoma.

## 2. Materials and Methods

### 2.1. Study Cohort

Three patients with UM were included in this feasibility study. The patients’ ages were 52, 65 and 79 years. All patients had presented with small uveal melanomas (T1N0M0) located on the posterior pole of the eye. The tumor size ranged from 7.8 to 8.0 mm in largest basal diameter and from 0.5 to 2.9 mm in thickness. In 2 cases, the clinical diagnosis was confirmed by a previous tumor biopsy examination. Written informed consent for the off-label use of the encircling band material and for the surgical procedure was obtained from all patients after the nature of the procedure was explained. Implantation was performed under general anesthesia.

### 2.2. Material and Surgical Procedure

The implantation of the silicone fiducial markers (SFMs) was performed 3–5 weeks prior to irradiation. The SFMs were manufactured from encircling bands (FCI Ophthalmics Inc., Pembroke, MA, USA) used for rhegmatogenous retinal detachment surgery. The material itself is CE-approved for use in retinal detachment surgery. All patients were informed about and agreed to the off-label use of the material as fiducial markers instead of TFMs.

**Figure 2:**
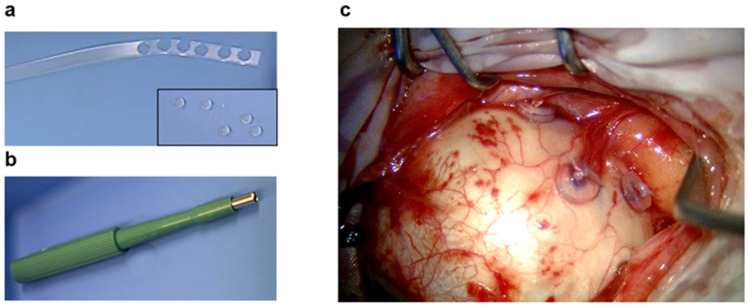
Photographs showing the encircling band and the fiducial markers punched out of the encircling band (**a**) and the punching instrument (**b**). Photograph showing the silicone fiducial markers fixed on the sclera with 4-0 Supramid (**c**). The intended marker position at the tumor margin was previously marked with a blue pen using fundoscopy and diaphanoscopy. The three clips mark the inferior, superior and anterior tumor margins (**c**).

With a 3 mm punch (KAI Europe GmbH, Solingen, Germany), markers from the silicone encircling band (0.75 mm in thickness, 3.5 mm in width) were prepared (Figure 2a,b) during the surgical procedure. The surgical procedure was the same for all patients. The conjunctiva was opened at the limbus in the quadrant containing the tumor and in the contralateral quadrant. Tagging sutures were inserted through the exposed rectus muscles. The sclera was inspected for evidence of extrascleral tumor extension. The tumor position and its margins were delineated via diaphanoscopic transillumination and indirect ophthalmoscopy. Tumor contours were marked with a blue pencil on the sclera (Figure 2c). The silicone markers were fixed close to the tumor margins (Figure 2c) on the sclera by non-absorbable surgical sutures (Supramid 4-0) (Figure 2c). Four to five markers were used for each case. One marker was fixed on the most anterior position, two were fixed at the lateral margins and one was fixed at the contralateral clock position. No attempt was made to mark the posterior margin of the tumor. During surgery, the marker placement was diagrammed in relation to the tumor base, and the distances between the fiducial markers as well as between the markers and the limbus were measured. The markers were not removed after radiotherapy.

### 2.3. Treatment Planning

For the treatment planning of robotic-guided stereotactic radiosurgery (SRS), all patients underwent pre-procedural MRI performed within 1 week before the radiotherapy, and treatment planning CT with an immobilization mask on the treatment day after retrobulbar injection in order to take the potential injection-induced bulbar shift into account. With the immobilized eye, accurate target delineation, treatment planning and administration of the radiation treatment were performed within two hours based on standard practice for out-patient robotic-guided SRS [20,21]. A retrobulbar block was performed with 2% prilocaine, bupivacaine and hyaluronidase.

The anterior borders of the gross tumor volume (GTV) were outlined by the SFMs. The optic nerve served as a posterior landmark. The distance between the optic nerve and the posterior margin of the tumor was previously measured and integrated into the drawn diagram and planning of the clinical target volume (CTV), which included the GTV plus 1 mm in all directions, except posterior, where it was 2 mm.

Treatment planning was based on standard practice according to known tumor dose–response models and normal tissue complication probabilities [20,21,22]. Accordingly, SRS was applied to a well-defined area with minimal exposure to the surrounding healthy tissue. All patients were irradiated with the robotic-based CyberKnife system (Accuray Inc., Sunnyvale, CA, USA). Patients were allowed to go home right after treatment after a final ophthalmology and radiation oncology consultation.

## 3. Results

### 3.1. Surgery-Related Findings

No adverse events or side effects were seen intra- or postoperatively, and the SFMs were well tolerated thereafter.

### 3.2. Imaging-Related Findings

**Figure 3:**
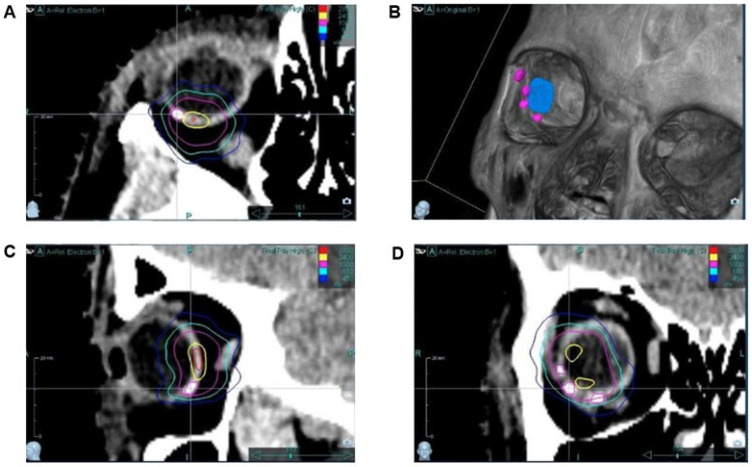
CT images of the silicone markers ((**A**) transverse; (**C**) sagittal; (**D**) coronal). Image (**B**) is the result of the VRT (volume rendering technique). Three of the markers were directly placed at the peripheral edges of the tumor, whereas the fourth marker was placed contralaterally. Tumor size (blue) was defined via fundus image, OCT and ultrasonography. In detail, tumor diameters and the distance of the tumor from the optic nerve and the macula measured with the 3 examination techniques were used to determine the tumor boundaries far from the markers. All markers could be clearly detected without scattering artifacts.

T1-weighted MRI sequences prior to the radiotherapy revealed hypodense areas at the position of the SFM. They were less visible than in the CT scan. In CT, the SFM showed a clear hyperdense signal without any scattering artifacts, which allowed the precise labeling of the target tumor volume (Figure 3). The maximum Hounsfield unit (HU) of the markers ranged from 120 to 140 HU as compared to the corpus vitreum (−10 to +10 HU) and the choroidea (40 to 60 HU) in the CT.

### 3.3. Stereotactic Radiosurgery

The gross tumor volumes (GTVs) and planning target volumes (PTV) for the three patients were 0.19/0.17/0.12cc and 0.44/0.45/0.28cc. The dose prescription was 23 Gy in 1 fraction for all three cases, resulting in a PTV D98%, PTV D2% and GTV D50% of 22.4/22.1/22.7 Gy, 30.4/31.9/32.0 Gy and 28.7/29.8/29.8 Gy based on International Commission on Radiation Units and Measurements (ICRU) Report 91 on standards for stereotactic radiotherapy reporting. The dose calculation was accurate (<0.1% difference) based on standard practice as the SFMs did not cause any streaking or canceling artifacts in the treatment planning CT. Furthermore, the silicon markers were not visible on the X-ray images during treatment delivery with the CyberKnife and hence did not affect the treatment delivery accuracy, as the CyberKnife uses the skull for beam steering during treatment.

### 3.4. Outcome

The main outcome of this study is to demonstrate the feasibility of the intraoperative preparation and use of SFMs to landmark the tumor and their visibility for treatment planning. The study was not intended to deliver any results on the tumoricidal effect, the radiation side effects and the development of metastatic disease. For these purposes, a longer clinical follow-up period after radiotherapy is needed.

## 4. Discussion

Herein, we present for the first time the successful use of silicone fiducial markers (SFMs) to guide UM treatment with robotic-guided stereotactic radiosurgery.

Large uveal melanomas usually present as sharply delineated, elevated spheroid lesions in CT and MRI. In these cases, there is no need to mark the tumor margins for accurate irradiation planning. In contrast, small, discoid intraocular tumors, larger tumors with flat extensions and UMs with adjacent serous retinal detachment need to be delineated to eliminate the geometrical inaccuracies in CT and MRI scans accordingly. For this purpose, tantalum fiducial markers (TFMs) were introduced almost 50 years ago by Gragoudas to indicate the target volume on CT scans and to help optimize the planning target volume for radiotherapy [15].

TFMs, which have been used for decades, are well tolerated but cause substantial scattering artifacts, reducing the quality of imaging and consequently treatment accuracy (Figure 1). Additionally, metal markers such as TFMs positioned in the radiation field can produce significant perturbations of the absorbed dose [23,24,25]. These limitations highlight the need for fiducial markers visible on CT and MRI scans that do not cause artifacts and that have a low absorbance of irradiating energy. Until now, there were only four publications dealing with this issue and investigating alternative material to be used as a fiducial marker. Zehetmayer et al. tested polymethyl methacrylate (PMMA) and barium-impregnated silicone markers [16]. Saini et al. evaluated ceramic material [17]. Daftari et al. compared titanium to tantalum markers [18], and Xu et al. tested carbon fiducial markers [19].

The PMMA marker tested by Zehetmayer et al. appeared to not be useful, since visibility in the CT scans was poor. The barium-impregnated markers, tested in two patients, instead showed good visibility without scattering artifacts in CT images [16]. The authors indicated that both PMMA and silicone are materials frequently used for intraocular implants in ophthalmology, but did not realize that silicone itself, depending on the density of the used material, might be sufficiently visible on X-ray images. Therefore, they used barium-impregnated silicone spheres normally utilized for performing artificial intravascular embolization [26]. A PubMed search using “Barium-impregnated silicone spheres” disclosed only four additional papers using the composite [27,28,29,30]. Though silicone is inert and has been used in many medical devices for decades, it appears that there are not many experiments of the combination with barium and the biocompatibility of this composite. There is also no follow-up study of the two implanted patients and no further attempts in this group, though their results appeared promising.

Similarly, there is no biocompatibility experiment with ceramic material placed on the bulbus. In fact, the fiducial markers with a ceramic core (zirconium oxide) and carbon coating used by Saini et al. were tested only in a phantom model. Though the markers were sufficiently visible in imaging, the analysis showed that small volumes behind the marker were severely under-dosed when irradiation was simulated [17].

A phantom model was also used to investigate titanium markers. Daftary et al. showed promising results concerning scattering artifacts [18]. The authors indicated that as the material density of titanium grade 2 metal (4.51 g/cm^3^) is lower compared to that of tantalum metal (16.4 g/cm^3^), perturbations of the absorbed dose will probably reduce with these markers. It should be noted here that our silicone markers only provide a density of 1.11 g/cm^3^. Since they cannot be detected in X-ray imaging, no relevant absorption and dose perturbations are to be expected during irradiation.

Since titanium is already used for medical implants, good biocompatibility might be expected. Nevertheless, so far, titanium markers placed on the sclera have not been evaluated in vivo, and no further publications pursuing this idea have been published yet.

Recently, Xu et al. reported the use of carbon fiducial markers in a case series of 11 patients [19]. The authors reported that the carbon fiducial markers had good visibility in MRI scans. However, in the figures shown in this publication, as well as in the pictures shown in their previous case report, the fiducials are barely visible in MRI. Additionally, both publications do not show any results related to X-ray imaging and CT, respectively. This imaging, however, is indispensable for radiotherapy planning and treatment. Since no further investigations were reported, we suppose that scattering artifacts might have been an issue. Additionally, 6 out of the 11 patients reported discomfort after the implantation of the carbon material. Though Xu et al. attribute this to the suturing material (5-0 nylon) and the anterior location of the marker, we did not have similar experiences with TFMs or with the novel markers used in this report.

Herein, we report a simple and ready-to-use approach that has the potential to replace tantalum for fiducial markers. We noted that encircling bands placed in the eye for retinal detachment surgery were detectable in MRI and CT scans. Additionally, there is longstanding evidence of the biocompatibility of implants that are made of silicone. Since encircling bands are CE-approved for suturing on the sclera and are obviously safe for use on human eyes, we opted to use this established medical product as a fiducial marker. This was undertaken with the consciousness that by punching out small clips from the band, we were modifying the product and using it off-label (Figure 2). A quite positive additional side effect during surgery was the easiness in handling the new fiducial markers compared to tantalum fiducial markers (Figure 2).

Starting with the first patient, we noted a quantum leap in delineation and treatment planning for robotic-guided stereotactic radiosurgery of the choroidal tumor. The clearly visible silicone markers in the CT images immediately enabled more precise target definition and treatment planning. Without scattering artifacts, the precise and fast identification of the marker edges and thus their exact localization were possible (Figure 3). An additional advantage of the new material is the reduced absorption of irradiation. The HU of the markers was only slightly elevated up to 140 HU, whereas TFMs have an HU of about 1500. In addition to artifacts, tantalum markers produce a significant perturbation of the applied dose, since absorption depends on the material density. The quite low material density of silicone significantly improves the dosimetry of radiation therapy treatment.

One limitation of the lower density, however, is the reduced visibility during stereoscopic X-ray imaging during treatment. The SFM cannot be used for eye localization and possible tracking during irradiation, and retrobulbar anesthesia is still necessary. During robotic SRS with the CyberKnife, the skull delineation is used as a surrogate for beam targeting. Nevertheless, further steps to introduce eye tracking systems in combination with SFMs are being developed.

## 5. Conclusions

In summary, we present for the first time a simple and convenient approach for fiducial markers in radiotherapy treatment planning for uveal melanoma. The silicone fiducial markers tested in our study were a relevant change and made a significant improvement to target delineation, treatment planning and the administration of stereotactic radiosurgery. Based on this very positive experience, more patients than described in this feasibility study have already been included in our daily clinical practice, in which silicone instead of tantalum fiducial markers are being used. However, this is only a preliminary report about feasibility, and further analysis of the radiation results of our patients with SFMs compared to the results of a TFM group will follow in the future.

In contrast to previously published studies on new fiducial markers, we employed a well-tolerated and long-approved material, which can be used right away.

## Data Availability

The raw data supporting the conclusions of this article will be made available by the authors on request.
